# The Multifaceted Nature of Weight-Related Self-Stigma: Validation of the Two-Factor Weight Bias Internalization Scale (WBIS-2F)

**DOI:** 10.3389/fpsyg.2019.00808

**Published:** 2019-04-16

**Authors:** Angela Meadows, Suzanne Higgs

**Affiliations:** ^1^School of Psychology, University of Exeter, Exeter, United Kingdom; ^2^School of Psychology, University of Birmingham, Birmingham, United Kingdom

**Keywords:** internalized weight stigma, internalized weight bias, self-stigma, anti-fat attitudes, factor analysis, Weight Bias Internalization Scale

## Abstract

**Background:**

Internalized weight stigma (IWS) is generally operationalized as self-devaluation due to weight in higher-weight individuals. The most commonly used measure of IWS, the Weight Bias Internalization Scale (WBIS), was developed from an original pool of 19 items. Item selection was guided by statistical techniques based upon an *a priori* hypothesized unidimensional factor structure. The resulting 11-item scale mostly assesses appearance-related attitudes, fear of stigma, affect, and desire for change, all of which may be a natural response to societal weight stigma, even in the absence of self-devaluation. Items pertaining to self-blame, stigma awareness, perceived legitimacy of weight stigma, and most items pertaining to self-worth, were excluded from the final scale. It is unclear whether an *a priori* assumption of multi-dimensionality would have produced different results.

**Methods:**

Exploratory and confirmatory factor analysis of the original 19-item questionnaire was conducted in 931 higher-weight individuals.

**Results:**

A 13-item two-factor structure was identified. Factor 1 comprised seven items that could be loosely conceived as weight-related distress. Factor 2 comprised six items, all of which pertained to weight-related self-worth. Tested individually, the six items making up the self-devaluation factor were an excellent fit for the data on all fit indices.

**Conclusion:**

IWS is a multi-dimensional construct. The two-factor WBIS (WBIS-2F) provides options to explore the relationships between different aspects of IWS and upstream and downstream variables. The Self-Devaluation subscale is suitable for standalone use when weight-related self-devaluation *per se* is the construct of interest.

## Introduction

Weight stigma can be broadly defined as exposure to negative attitudes, behaviors, or structural indignities that befall higher-weight individuals because of their weight or size. Higher-weight individuals experience weight stigma in practically every domain of daily life (Puhl and King, 2013). In addition to being stigmatized by others, some individuals internalize society’s anti-fat attitudes and stereotypes – that is, they devalue themselves because of their weight, with concomitant detriment to their self-worth and social identity (Hunger et al., 2015). Internalized weight stigma (IWS) has been linked with a wide range of negative health outcomes, including mood disorders, psychological distress, worse body image, lower self-esteem, poorer health-related quality of life, metabolic dysfunction, disordered eating, avoidance of exercise, and social isolation and experiential avoidance (for a review, see Pearl and Puhl, 2018). Importantly, IWS appears to be an important mediator in the relationship between experienced stigma and maladaptive coping behaviors including disordered eating (Durso et al., 2012; O’Brien et al., 2016; Meadows and Higgs, unpublished) and reduced physical activity (Pearl et al., 2015), and between BMI and health-related quality of life (Lillis et al., 2011). IWS also moderated the relationship between BMI and physical health-related quality of life in a sample of 81 higher-weight women recruited from weight-related Internet sites, such that the negative association was observed only in those individuals with high levels of IWS (Latner et al., 2014). Thus, IWS appears to be a critical consideration in understanding negative health outcomes in higher-weight individuals.

### Operationalizing Internalized Weight Stigma

One of the major issues facing researchers of IWS is that of operationalization – that is, how the construct is defined. IWS is most commonly defined as not just awareness, or even endorsement, of negative stereotypes, but also as applying those negative attributes to yourself, *and* subsequently devaluing yourself because of it (Durso and Latner, 2008). For example, while IWS does include a component of negative appearance evaluation, this is specific to facets of body image related to weight. Additionally, there is a strong element of self-blame involved in IWS. For example, while one might have poor body image related to a specific body part, such as height, or a disliked facial feature, this is unlikely to be tainted by a belief that one is to blame for that aspect of one’s appearance. Similarly, self-esteem that is specific to the domain of weight does not preclude higher self-worth in other domains, and vice versa. Finally, IWS is a self-directed attitude, whereas anti-fat attitudes generally pertain to evaluations of fat others. Thus, IWS is related to, but distinct from, the constructs of body image, self-esteem, and attitudes toward other high-weight individuals (Durso and Latner, 2008; Carels and Musher-Eizenman, 2010; Carels et al., 2013). Unusually among marginalized groups (Tajfel and Turner, 1979; Crandall et al., 2000; Dasgupta, 2004), there appears to be little protective ingroup bias among higher-weight individuals; that is, fat people are as likely to hold negative explicit and implicit anti-fat attitudes as are slimmer people (Crandall and Biernat, 1990; Crandall, 1994; Rudman et al., 2002; Wang et al., 2004; Schwartz et al., 2006). Further, negative attitudes toward other higher-weight individuals are not necessarily reflected in one’s views of oneself (Carels et al., 2011). In a study of 53 higher-weight adults enrolled in a weight-loss intervention, participants demonstrated high levels of both explicit and implicit negative attitudes toward higher-weight individuals in general; however, implicit attitudes testing suggested that despite their self-assigned “overweight” status and their participation in a weight-loss intervention, they saw themselves as significantly thinner, better, and more attractive, active, disciplined, and likely to eat healthily than fat others (Carels et al., 2011). In fact, it appears that many fat people do not self-identify as fat – perhaps envisioning themselves as thin people in merely temporarily fat bodies (Quinn and Crocker, 1998; Murray, 2005; Kyrölä and Harjunen, 2017).

### Measures of Internalized Weight Stigma

To date, three validated measures of IWS have been published, all using slightly different conceptualizations of the construct. Although not formally depicted as a measure of IWS, the Weight- and Body-Related Shame and Guilt Scale (WEB-SG; Conradt et al., 2007), assesses feelings of shame at one’s size and guilt at failing to engage in supposed weight-changing behaviors. A typical item on the Guilt subscale is, “When I can’t get a grip on my weight, I blame myself.” Lewis (1971) proposed that the related emotions of shame and guilt differ primarily in the role of the self: whereas guilt represents a state of negative affect relating to, for example, a specific deviant behavior (e.g., lying, stealing), with the behavior being the focus of judgment, shame represents a more trait-level attribution to negative self-worth, whereby moral transgressions are transmitted into a global devalued self (Lewis, 1971; Tangney et al., 1996). Thus, shame, rather than guilt, should be more aligned with the conceptualization of IWS as a self-devaluation status. More recent conceptualizations of the construct of shame have identified two aspects of shame – one that concerns global self-defect and one relating to appraisal of condemnation by others (Gausel and Leach, 2011; Gausel et al., 2016). Most of the six items on the Shame subscale of the WEB-SG refer specifically to anticipated rejection by others, for example, “When I am in a situation where others can see my body (e.g., pool, changing room), I feel ashamed.” Thus, this subscale primarily captures perceptions of damaged social image, rather than specific self-defect. Although both subscales explained additional variance in scores on body self-acceptance, depressive symptoms, and self-esteem, beyond that accounted for by shame and guilt related specifically to eating (Conradt et al., 2007), a study involving a weight-diverse sample of Canadian young adults found that the Shame, but not Guilt, subscale, mediated the relationship between objective measurements of weight status (BMI, skinfolds, and waist circumference) and global self-esteem (Pila et al., 2015).

A second validated measure of IWS is the Weight Self-Stigma Questionnaire (WSSQ; Lillis et al., 2010). The WSSQ comprises two subscales, which differentiate between self-devaluation and fear of being stigmatized by others. The Self-Devaluation subscale assesses guilt, shame, and self-blame with respect to body weight, and includes items such as, “I feel guilty because of my weight problems,” and “I caused my weight problems.” Three of the six items relate to a global self-defect, but all pertain to willpower, for example, “I became overweight because I’m a weak person.” No items relate to other aspects of a devalued self. The Fear of Enacted Stigma subscale assesses worries about being stigmatized by others because of weight, for example, “Others are ashamed to be around me because of my weight,” and “Others will think I lack self-control because of my weight problems.” It should be noted, then, that although the authors of the scale denoted this subscale as “fear of stigma,” the subscale could also be characterized as anticipation or expectation of weight stigma – that is, fear of rejection and feelings of inferiority due to other-condemnation, and overlaps considerably with the Shame subscale of the WEB-SG. It could be argued that devaluing oneself due to a stigmatized characteristic may lead to expectations that others will do the same, but it is not a necessary pre-requisite (Link et al., 2015). Nevertheless, using the WSSQ, Almenara et al. (2017) reported that self-devaluation, but not fear of stigma, was associated with recent dietary restraint and eating and weight concerns in higher-weight women. Thus, while studies using these measures are clearly telling us *something* about the relationship between weight-related self-beliefs and health and behavioral outcomes, interpretation of these findings is constrained by the lack of clear agreement on the theoretical underpinnings of the construct.

The third validated measure of IWS is the Weight Bias Internalization Scale (WBIS; Durso and Latner, 2008). The WBIS was developed from an original pool of 19 items encompassing several potential aspects of weight-related self-stigma, including appearance-related attitudes, social status, fear of being stigmatized by others, affective impact of weight status, desire for change, and weight stigma awareness and perceived legitimacy of weight stigma. Item selection for the final scale was guided by statistical techniques based upon a hypothesized unidimensional construct, producing a final scale comprising eleven items. These items mostly assess attitudes related to appearance, fear of stigma, affect, and desire for change. Notably, all items pertaining to self-blame, stigma awareness, and perceived legitimacy, and several of the items pertaining to self-worth, were excluded from the final scale.

Given that the key underlying concept involved in IWS is one of self-devaluation, it could be questioned whether the standard WBIS, here denoted WBIS-11 for clarity, optimally captures this construct. Although the WBIS-11 is widely used, Schvey et al. (2013) used the full 19-item version of the WBIS (WBIS-19) in an online sample of 656 overweight and obese adults and the scale demonstrated excellent internal reliability and convergent and discriminant validity. Scores on the WBIS-19 were significantly correlated with eating disordered cognition and behavior, history of high-weight status, weight cycling, and depressive symptoms, even after controlling for BMI. Additionally, WBIS-19 scores discriminated between participants who engaged in binge/purge behavior and those without eating pathology (Schvey et al., 2013). However, the factor structure of the WBIS-19 has not been tested. The aim of the present study was to conduct the first exploratory and confirmatory factor analysis of the WBIS-19 using split samples from the same population of higher-weight individuals to establish its latent variable structure. This analysis was conducted as part of a broader study on individual differences in response to weight stigma.

## Materials and Methods

### Sample

A purposive recruitment strategy was implemented, designed to provide a sample likely to have a range of views on the acceptability of societal weight stigma, both positive and negative emotions about their own body weight, and to differ in their levels of fat identity. As such, adult participants (age 18–69 years) who self-identified as “overweight,” “obese,” or “fat”^1^ were recruited to complete an anonymous online survey on the “Life experiences of overweight individuals,” and invitations to participate in the survey were posted on social media and Internet forums related to weight, weight-loss, health, nutrition, fitness, plus-size fashion, and the size acceptance movement.

The choice to use these three terms to describe weight status was a deliberate one. Higher-weight individuals have different preferences for the terminology used to describe their bodies, often finding one or more of the terms offensive. For example, members of the “size acceptance” community – one of the groups targeted in the recruitment process – prefer the word “fat” and dislike medicalized terms of body weight (Meadows and Daníelsdóttir, 2016). Although evidence suggests that various weight-related terminology carries different meanings to different people, including more normative terms with medically designated definition such as “overweight” and “obese” (Vartanian, 2010; Brochu and Esses, 2011; Ellis et al., 2014), this approach augments the diversified recruitment strategy, helping to address the limitations of non-generalizability of findings from, for example, treatment-seeking populations, and also increasing the likelihood of attaining sufficient variation on the measure of interest to conduct reliable psychometric testing.

A two-step inclusion criteria was used, involving both self-classification of higher-weight status, and having a BMI consistent with the standard definitions of high-weight status – that is, a self-reported height and weight producing a BMI greater than or equal to 25 kg/m^2^. Extensive evidence testifies to the fact that self-identification of body size is either an equally or more consistent predictor of cognitive, affective, and behavioral correlates than is objective BMI (Major et al., 2014; Lee and Dedrick, 2016; Lin et al., 2018). However, this double-classification method has been used previously as a more conservative sample selection procedure (Durso et al., 2012; Pearl and Puhl, 2016).

The survey was conducted using a dedicated survey platform^2^. After providing consent, participants completed a series of questionnaires and provided demographic data. All participants were entered into a prize draw to win one of two £50 Amazon voucher (or local equivalent). The study was approved by the University of Birmingham Ethical Review Committee.

### Measures

Internalized weight stigma was measured with the WBIS-19 (Durso and Latner, 2008) (see Table 1). Items were scored on a seven-point Likert scale ranging from 1 (*strongly disagree*) to 7 (*strongly agree*), with higher scores indicating greater weight-related self-stigma. Internal reliability of the WBIS-19 was 0.92. Participants were asked to provide age, gender, and ethnicity, and to report height and weight measurements, which were used to calculate BMI. The option to decline to answer any of these questions was provided.^3^

**Table 1:** Exploratory factor analysis of WBIS-19.

Item	*F*1	*F*2	*F*3
1. It is my fault that I am overweight	0.48	0.46	–
2^∗^. As an overweight person, I feel that I am just as competent as anyone^a^	–	–	0.74
3. I am less attractive than most other people because of my weight^a^	0.69	–	–
4. I feel anxious about being overweight because of what people might think of me^a^	0.72	–	–
5. I wish I could drastically change my weight^a^	0.84	–	–
6. If only I had more willpower, I would not be the weight that I am^a^	0.59	0.48	–
7. Whenever I think a lot about being overweight, I feel depressed^a^	0.79	–	–
8^∗^. I feel that being overweight does not interfere with my ability to be a good and decent person	–	–	0.55
9. I hate myself for being overweight^a^	0.76	–	–
10. My weight is a major way that I judge my value as a person^a^	0.61	–	–
11. I do not feel that I deserve to have a really fulfilling social life as long as I am overweight^a^	–	–	0.47
12^∗^. I am OK being the weight that I am^a^	0.74	–	–
13^∗^. As an overweight person, I feel that I am just as deserving of respect as anyone	–	–	0.78
14^∗^. It really bothers me that people look down on overweight people	–	0.74	–
15. Because I am overweight, I do not feel like my true self	0.76	–	–
16^∗^. I feel that being an overweight person does not make me unworthy of a loving relationship	–	–	0.34
17. Because of my weight, I do not understand how anyone attractive would want to date me^a^	0.61	–	–
18^∗^. I believe that society’s prejudice against overweight people is unfair	–	0.70	–
19. If other people do not treat me with respect, I should put up with it because of my weight	–	–	0.61
Internal reliability^b^	0.93	0.80^c^	0.77

*N = 481. Standardized factor loadings displayed. WBIS = Weight Bias Internalization Scale. ^∗^Items marked with an asterisk are reverse-scored. ^*a*^Items included in standard WBIS-11. ^*b*^Internal reliability statistic is Cronbach’s α except for two-item *F*2, which is Spearman–Brown coefficient. ^*c*^Items 1 and 6 not included – assumed to load onto *F*1 only. Alpha with items 1 and 6 included = 0.76.*

### Handling of Missing Values

Fifty-one participants (5.5%) were missing height and/or weight information such that BMI could not be computed. Three participants (0.003%) had missing responses on one (*n* = 2) or two (*n* = 1) items on the WBIS-19. Missing values analysis indicated no overall pattern of missingness, Little’s MCAR test χ^2^(75) = 91.7, *p* = 0.09, indicating that these data were missing completely at random. Independent samples *t*-tests confirmed no response differences between participants with or without BMI data available. As BMI was collected predominantly for descriptive purposes, and was not included in the hypothesized model, missing BMI values were not imputed. Given the very low prevalence of missing data on the WBIS-19, no imputation was used and factor analyses were conducted with listwise deletion. Missing values on demographic variables (race/ethnicity 8.1%, age, geographic location, education, and profession all <3.8%) were also not imputed.

### Data Analysis

The data were split randomly into two groups, each including approximately 50% of cases. Exploratory factor analysis was conducted on one half of the data (*N* = 481), using principal axis factoring and direct oblimin rotation with Kaiser normalization. It was stipulated that item factor loadings >0.3 represented a substantive contribution of the item to a factor. Given the large sample size, factor extraction decisions were based on the scree plot, rather than eigenvalues (Field, 2013). Internal reliability was calculated for each derived factor.

Confirmatory factor analysis was conducted with the other half of the data (*N* = 450) using maximum likelihood estimation. Model fit was assessed using χ^2^ values, comparative fit index (CFI), and standardized root-mean-squared residuals (SRMR). Cut-off values of 0.95 for the CFI and 0.08 for SRMR, respectively, are generally considered to indicate a relatively good fit of the hypothesized model to the observed data (Hu and Bentler, 1999). However, CFI tends to decline with increasing number of indicators in the model (Kenny and McCoach, 2003). In the present analysis, the maximum number of variables per factor was 19, thus, following Chen et al. (2012), a less stringent cut-off of 0.90 was used for the CFI to indicate goodness of fit in models with higher number of factor loadings. Additionally, as the sample size approached 500, root-mean-square error of approximation (RMSEA) and its 90% confidence interval would be more reliable than in smaller samples (Hu and Bentler, 1999), and was included as an additional measure of model fit. The RMSEA is an indicator of the proportion of variance not explained in the model. A value of RMSEA of 0.06 or lower is considered indicative of good model fit, below 0.08 a reasonable fit, and values above 0.10 indicate poor model fit (Browne and Cudeck, 1992; Hu and Bentler, 1999). Model comparison (i.e., selection of superior models) was assessed using fit indices (CFI, RMSEA, SRMR) plus χ^2^ difference tests. A reduction in χ^2^ greater than the critical value for the change in degrees of freedom indicates a significantly better model fit. Confirmatory factor analysis was conducted using Mplus version 8 (Muthén and Muthén, 1998–2017). All other analyses were conducted using IBM SPSS for Mac v25.

## Results

### Sample Characteristics

A total of 1154 participants began the study and 963 (83.4%) completed it. Twenty-six participants (2.7%) had a BMI less than 25 kg/m^2^ based on self-reported height and weight and these participants were excluded from subsequent analyses. Five participants were aged over 69 years (70–80 years) and one was aged 17. These participants fell outside the age range specified in the approved ethical application for this study and were also excluded. The final sample size was therefore 931.

The sample was predominantly female (85.5%; 9.7% male, 1.9% other, 2.9% missing) and White (83.7%; 1.9% Black, 1.5% Hispanic, 1.2% Asian, 2.1% multi-racial, 8.2% other, 8.1% missing). Age range was 18–69 years (*M* = 40.2, *SD* = 11.4; 3.8% missing), and BMI range was 25.0–95.0 kg/m^2^ (*M* = 40.2, *SD* = 10.8). Further breakdown of BMI distribution indicated 14.1% had BMI between 25.0–29.9 kg/m^2^, 21.4% between 30.0–34.9 kg/m^2^, 17.9% between 35.0–39.9 kg/m^2^, 27.8% between 40.0–49.9 kg/m^2^, and 13.3% had BMI greater than or equal to 50.0 kg/m^2^. Just over one-third were living in the United Kingdom and just over a half in North America – no other region accounted for more than 5% of the sample. Participants were also highly educated, with three-quarters having a college degree or higher, and 61.3% listed their occupation as managerial, administrative, or professional; 9.5% were students, 5.2% unemployed, 20.9% other, 3.2% missing.

WBIS-19 scores were normally distributed (minimum = 1.1, maximum = 6.4, *M* = 3.6, *SD* = 1.1) with low skewness (-0.174, *SE* = 0.08) and kurtosis (-0.554, *SE* = 0.16), indicating a good distribution of IWS scores. Small but significant correlations were observed between WBIS-19 and BMI (*r* = -0.13, *p* < 0.00).

### Exploratory Factor Analysis

Exploratory factor analysis based on a random half (approximate) of the sample (*N* = 481) suggested a three-factor structure for the WBIS-19, explaining 54.8% of the total variance (see Table 1 for individual items and factor loadings; see Supplementary Material for scree plots). The first factor (*F*1) included 11 items and was almost identical to the standard WBIS-11. While these items appear conceptually diverse, this factor could be loosely conceived as “weight-related distress” – negative cognitive and affective states resulting from weight status, for whatever reason, whether related to how you look, how others treat you, if you blame yourself for getting that way, and so on. Corrected item-total correlations for the 11-item factor ranged from 0.55 to 0.81. The second factor (*F*2) initially comprised four items, two pertaining to the perceived legitimacy of anti-fat attitudes – “It really bothers me that people look down on overweight people” and “I believe that society’s prejudice against overweight people is unfair” (items 14 and 18 in the original WBIS-19), and two pertaining to self-blame – “It’s my fault that I’m overweight” and “If only I had more willpower, I wouldn’t be the weight I am” – that had similar factor loadings across both *F*1 and *F*2 (items 1 and 6 on the original WBIS-19. The third factor (*F*3) comprised six items, all of which pertained to weight-related self-worth, and this factor was labeled “weight-related self-devaluation.” Corrected item-total correlations for this six-item factor ranged from 0.36 to 0.66. *F*1 and *F*3 correlated 0.530, but *F*2 did not correlate strongly with either of the other factors (*r*s = 0.146 and 0.225, respectively). Additionally, *F*2 contributed the least proportion of total variance explained (rotated sum of squared loadings *F*1 = 6.94, *F*2 = 2.10, *F*3 = 4.91).

Of the four items loading onto F2, the two items pertaining to perceived legitimacy did not correlate strongly with any of the other items on the scale (15 and 14 correlation coefficients, respectively, below 0.3). In contrast, the items pertaining to self-blame correlated greater than 0.3 with 11 of the remaining 17 items. Given the imbalance of the number of items across the three factors, the low correlation of F2 with the other two factors, the very low correlations between items 14 and 18 and the remaining 17 items, and the relatively small contribution to the total variance explained, it was decided to delete the two items pertaining to perceived legitimacy but to retain items 1 and 6 at this stage. Thus, the analysis was re-run with the remaining 17 items.^4^

EFA of the WBIS-17 again produced three factors, explaining 56.0% of the total variance, with the items pertaining to self-blame no longer cross-loading, but now loading uniquely onto their own factor. However, as these items have previously loaded onto *F*1, the EFA was re-run pre-specifying a two-factor extraction. This analysis produced a clear pattern of factor loadings, with 11 items loading onto a weight-related distress factor (including the items pertaining to self-blame), and six onto a weight-related self-devaluation factor. Internal reliability of the weight-related distress factor was 0.926. Internal reliability of the weight-related self-devaluation factor was 0.768. Item 16 – “I feel that being an overweight person does not make me unworthy of a loving relationship” (reverse-scored) had a lower item-total correlation than the other items on the factor (0.360), and its deletion would have increased the internal reliability to 0.794. However, given the relatively small size of this improvement, that deletion would also have a large impact on scale variance – reducing it from 39.4 to 27.6, and the smaller number of items on this factor, a decision was made to retain this item at this stage.

### Confirmatory Factor Analysis

Confirmatory factor analysis using the remainder of the sample (*N* = 450) tested the 17-item two-factor model identified by exploratory analysis. The factors were allowed to covary. The two-factor structure of the WBIS-17 was a poor fit to the data (Table 2).^5^ As the factors remained unbalanced in terms of item number, with *F*1 comprising 11 items and *F*2 only 6, an alternative 15-item structure was tested, which involved removal from *F*1 of the two items pertaining to self-blame that had loaded onto their own factor when the number of factors to be extracted was not pre-specified. The resulting two-factor WBIS-15 was an acceptable fit for the data and superior to the WBIS-17 model on all fit indices. Investigation of modification indices (MIs) indicated nine pairs of items with values above 10. The highest of these was for item 12 – “I am OK being the weight I am” (reverse-scored) and item 5 – “I wish I could drastically change my weight,” MI = 77.8. Item 5 had slightly higher estimated factor loading and estimate/standard error, and was slightly more strongly correlated with other items on the scale; thus, item 12 was deleted and the CFA re-run.

**Table 2:** Confirmatory factor analysis of two-factor WBIS-17.

Model	χ^2^	Df	RMSEA [90% CI]	CFI	SRMR
Two-factor WBIS-17	694	118	0.104 [0.097,0.112]	0.857	0.061
Two-factor WBIS-15	365	89	0.083 [0.074,0.092]	0.920	0.049
Two-factor WBIS-14	259	76	0.073 [0.063,0.083]	0.941	0.047
Two-factor WBIS-13 (WBIS-2F)	180	64	0.064 [0.053,0.075]	0.957	0.045
*WBIS-2F Subscales*					
Weight-related distress	42.1^a^	14	0.067 [0.044,0.090]	0.984	0.022
Weight-related self-devaluation	16.2^b^	9	0.042 [0.000,0.075]	0.988	0.022
WBIS-11 (standard scale)	285	44	0.110 [0.098,0.123]	0.917	0.046

*N = 450. Compared with the original 19 items, WBIS-17 excludes items 14 and 18; WBIS-15 further excludes items 1 and 6; WBIS-14 further excludes item 12; WBIS-13 further excludes item 3. CFI = comparative fit index; CI = confidence interval; *df* = degrees of freedom; RMSEA = root-mean-square error of approximation; SRMR = standardized root-mean-squared residual; WBIS = Weight Bias Internalization Scale. All χ^2^*p* < 0.0000 unless otherwise stated. ^*a*^*p* = 0.0001. ^*b*^*p* = 0.06.*

The resulting WBIS-14 was a good fit to the data. Five pairs of items had MIs above 10, the largest of which was for item 3 – “I am less attractive than most other people because of my weight” and item 17 – “Because of my weight, I don’t understand how anyone attractive would want to date me,” MI = 38.4. Item 17 had slightly higher factor loading and estimate/standard error value. Additionally, while item 3 could be described as reflecting body image, item 17 additionally includes a component of self-worth. Thus, item 3 was removed and the CFA repeated.

**FIGURE 1:**
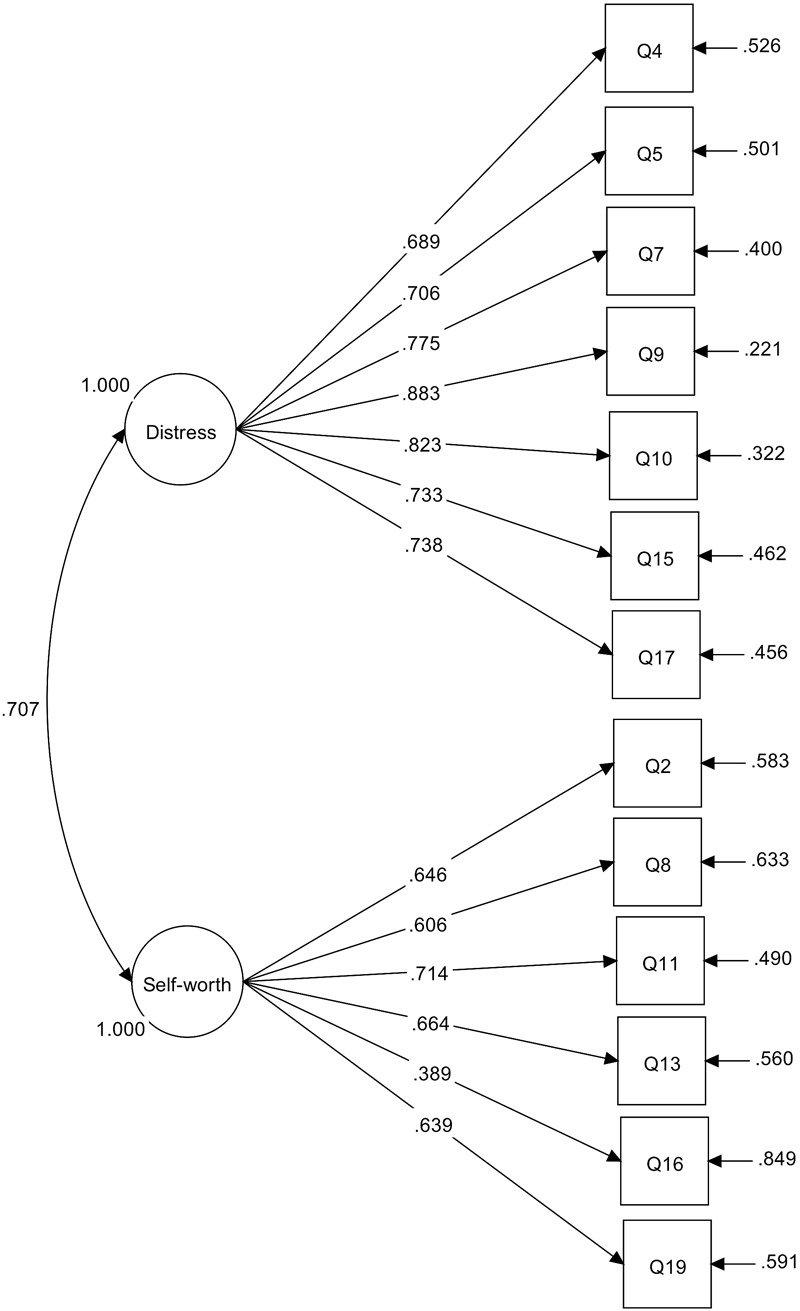
Measurement model for WBIS-2F subscales. Standardized parameter estimates are shown, all *p* < 0.000. Item numbers refer to numbering in original 19-item WBIS (Durso and Latner, 2008).

The resulting two-factor WBIS-13 (WBIS-2F) was a very good fit to the data. Three MIs had a value above 10, but none involved overlap on face validity, and no further changes were made. The final 13-item scale therefore included a seven-item weight-related distress factor and a six-item weight-related self-devaluation factor (Figure 1).^6^ Additionally, the two subscales individually were a good (weight-related distress) to excellent (weight-related self-devaluation) fit for the data.^7^ By comparison, the standard unidimensional WBIS-11 was an acceptable (CFI, SRMR) to poor (RMSEA) fit for the data.

Using the full sample, scores on the two factors indicated higher levels of weight-related distress (*M* = 4.6, *SD* = 1.5) than weight-related self-devaluation (*M* = 2.1, *SD* = 1.0). Weight-related distress and self-devaluation were moderately correlated (*r* = 0.58, *p* < 0.00). Cronbach’s α for the two subscales was 0.910 and 0.763, respectively.

## Discussion

The present study utilized a large, diverse sample of non-treatment-seeking higher-weight individuals to conduct the first examination of the latent variable structure of the original pool of 19 items that produced the standard WBIS-11 (Durso and Latner, 2008). As noted above, the WBIS-11 was derived based upon the assumption that the construct of IWS was unidimensional. However, the items in the resulting 11-item scale appear to represent a combination of underlying concepts, including fear of how one might be judged by others, desire for change, and psychological distress, all of which may be a natural response to societal weight stigma, even in the absence of self-devaluation. Removing the assumption of unidimensionality resulted in a three-factor scale, the dimensions representing weight-related self-devaluation, weight-related distress, and perceived legitimacy of weight stigma. Interestingly, item 1 on the standard WBIS-11 (Q2 on the WBIS-19: “As an overweight person, I feel that I am just as competent as anyone”), which has often been found to have low item-total correlation with the remaining 10 items and is frequently dropped from the scale (Durso et al., 2016; Lee and Dedrick, 2016), here loaded onto the self-devaluation factor rather than the weight-related distress factor that closely resembles the standard WBIS-11.

Only two of the original 19 items described beliefs about perceived legitimacy of weight stigma and loaded onto an independent factor that did not correlate strongly with the others, despite the face validity of the construct for a measure of weight-related self-stigma. It is possible that individuals may have strong views about social-justice issues, independent of their thoughts and feelings about their own bodies. Similarly, two items pertaining to self-blame loaded onto a separate factor when the number of factors extracted was not constrained. Forcing a two-factor extraction to avoid another two-item factor, these items loaded acceptably onto the weight-related distress factor, but confirmatory factor analysis indicated that the model was a better fit to the data without them. Thus, both perceived legitimacy of weight stigma and perceived controllability of weight, while related to IWS, can be excluded from a parsimonious scale that comprises weight-related distress and weight-related self-worth.

Confirmatory factory analysis indicated that a two-factor 13-item WBIS (WBIS-2F) was a good fit to the data. Exploring participants’ scale scores, the fact that scores on the weight-related distress subscale were notably higher than those on the self-devaluation subscale provides support for the contention that these items are measuring something different to self-devaluation. It could be that the weight-related self-devaluation subscale provides a true measure of perceived internal worthiness, or lack thereof, whereas the weight-related distress subscale represents feelings and thoughts associated with fears of not fitting into society. Thus, this multi-dimensional scale structure provides a more nuanced representation of internalized weight-related cognitions and affect than does the standard WBIS-11.

The Weight-Related Self-Devaluation factor provided the best statistical fit to the data when tested individually, suggesting that these six items could be used as a standalone scale when the research question focuses specifically on weight-related self-worth. Although the Weight-Related Distress factor was a good fit for the data when tested independently, its similarity to the standard WBIS-11 may negate any benefit of using it in this way, and it may be preferable to continue use of the standard WBIS-11 when a broader conceptualization of weight-related self-stigma is of interest, in order to retain comparability with the extant literature. Additionally, a small number of items on the weight-related distress subscale do refer to self-worth. Future work on IWS may benefit from revisiting this construct, using a larger number of pool items, possibly generated with input from the target population, a large, diverse sample, and a thorough psychometric validation of the resulting scale(s).

This study has a number of strengths. First, the large sample size permitted cross validation of the factor structure in two groups of non-treatment-seeking higher-weight individuals. Second, the participants represented a good range of body sizes across the higher-weight spectrum and diverse weight-related attitudes. However, there are also a number of limitations. The sample lacked gender, ethnic, and geographic diversity, which precluded testing of measurement invariance and latent mean differences on factor scores across groups. Subjects may have been prone to social desirability responding, particularly with regard to the perceived legitimacy questions. As no measure of social desirability responding was used, it was not possible to test this. Additionally, if the WBIS-2F and its subscales are to be used in future research, further assessment of the psychometric properties of the scale(s) will be needed.

## Conclusion

Internalized weight stigma may usefully be conceptualized as a multi-dimensional construct, encompassing both weight-related self-devaluation and more generalized cognitions and emotions related to living in a high-weight body in an anti-fat environment. The two-factor WBIS-2F could be used to explore the relationship between specific aspects of weight-related self-stigma and other upstream and downstream variables. Additionally, the six-item Self-Devaluation subscale aligns most closely with the original conceptualization of IWS, as a measure of reduced weight-related self-worth. This scale is suitable for standalone use when self-devaluation is the construct of interest.

## Ethics Statement

This study was carried out with written informed consent from all subjects in accordance with the Declaration of Helsinki. The protocol was approved by the University of Birmingham Ethical Review Committee.

## Author Contributions

AM conceived the study and was responsible for data acquisition and analysis. AM and SH contributed to the design of the study and interpretation of the data. AM drafted the initial version of the manuscript. Both authors were involved in critical revision of the manuscript, approved the final manuscript, and agreed to be accountable for all aspects of the work.

## Conflict of Interest Statement

The authors declare that the research was conducted in the absence of any commercial or financial relationships that could be construed as a potential conflict of interest.

## Footnotes

1 The word “obese” was added to recruitment materials in the present study as a result of a number of emails received from potential participants in a previous study that recruited “overweight” individuals. Respondents had queried whether they were eligible to participate if they were “obese” rather than “overweight.”

2 http://Qualtrics.com

3 While not included in the present analyses, other measures included in the full study were: Rosenberg Self-Esteem Scale (Rosenberg, 1965); Stigma Consciousness Questionnaire (Pinel, 1999); Multicomponent Ingroup Identification Scale (Leach et al., 2008); three items from the Anti-Fat Attitudes Questionnaire-Revised Willpower subscale (Quinn and Crocker, 1999); Stigma Resistance Scale and Perceived Legitimacy of Anti-Fat Discrimination – created for study and available from authors. These measures correlated with WBIS-19 scores in the expected directions, and internal reliability and validity of all questionnaires was high.

4 To determine whether the two items on *F*2 could be incorporated into a two-factor structure, EFA was repeated on the WBIS-19 but with a pre-specified two-factor extraction. This resulted in a confused pattern matrix with the two items loading onto one factor, 12 items onto the other factor, four items cross-loading across both factors, and one item not loading onto either factor above a loading value of 0.3.

5 A unidimensional 17-item model was also a poor fit to the data: χ^2^(119) = 936, RMSEA = 0.124 (90% CI = 0.116, 0.131], CFI = 0.797, SRMR = 0.079.

6 A unidimensional 13-item scale was not a good fit for the data: χ^2^(65) = 400, RMSEA = 0.107 (0.097, 0.117), CFI = 0.867, SRMR = 0.074.

7 Deleting item 16 from the weight-related self-devaluation factor, despite its lower factor loading (standardized loading 0.393) and relatively low proportion of variance explained (*R*^2^ = 0.154), did not improve the model and produced deterioration in several fit indices; thus, this item was retained.
