# Supplementary material for: The Multifaceted Nature of Weight-Related Self-Stigma: Validation of the Two-Factor Weight Bias Internalization Scale (WBIS-2F)
Source: Front Psychol. 2019 Apr 16;10:808. doi: 10.3389/fpsyg.2019.00808 (PMC6477068; doi:10.3389/fpsyg.2019.00808)
Supplement: Supplementary file 1 [file Data_Sheet_1.PDF]

Supplementary Material

The multifaceted nature of weight-related self-stigma: Validation of the Two-Factor Weight Bias Internalization Scale (WBIS-2F)

Angela Meadows*, Suzanne Higgs

*** Correspondence:** Angela Meadows: drameadows@gmail.com

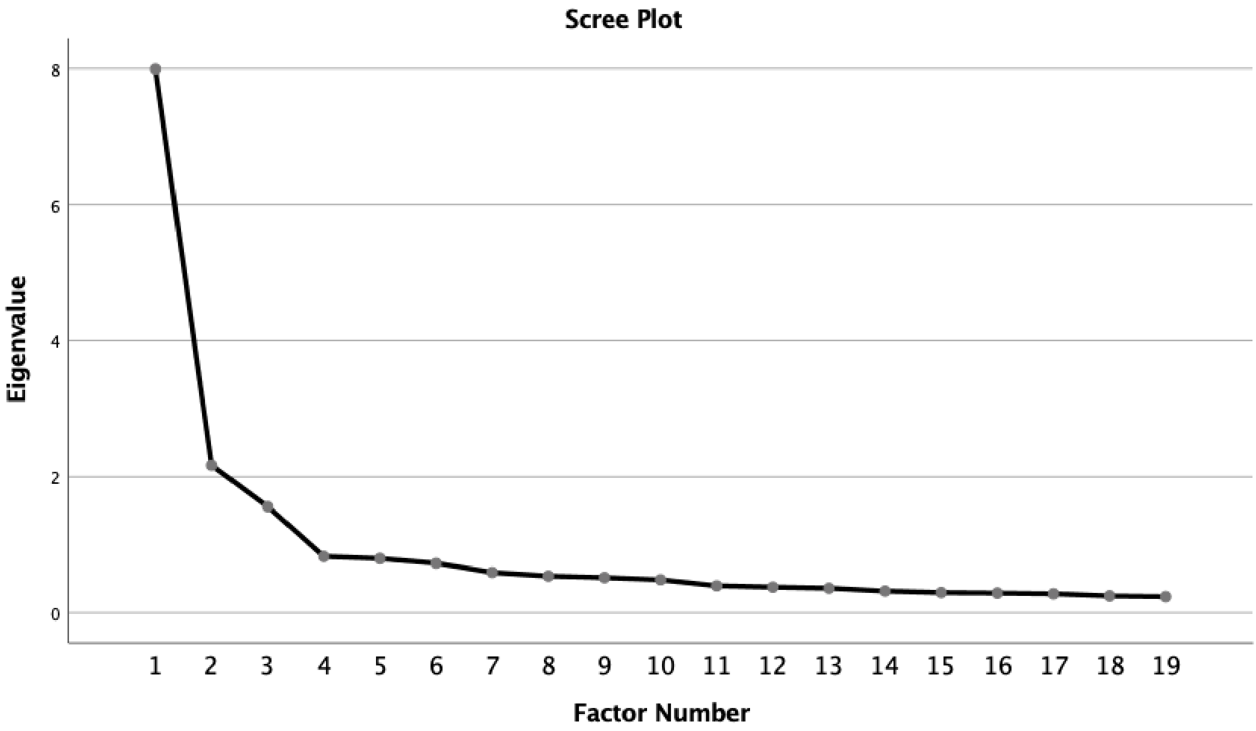

Supplementary Figure 1. Scree plot from initial exploratory factor analysis of WBIS-19 (N = 481). Three factors extracted explaining 54.8% of total variance. Rotated sum of squared loadings F1 = 6.94, F2 = 2.10, F3 = 4.91.

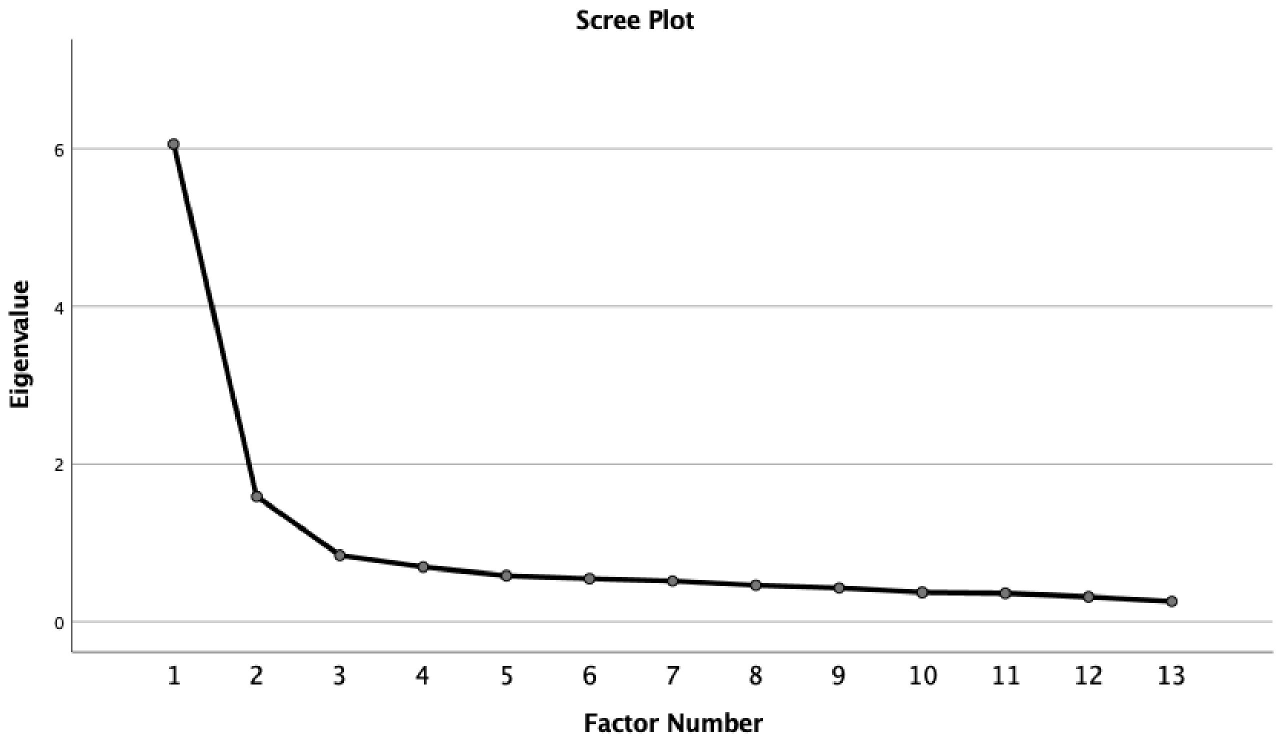

Supplementary Figure 2. Scree plot from exploratory factor analysis of WBIS-13 (*N* = 481). Two factors extracted explaining 52.7% of total variance. Rotated sum of squared loadings F1 = 5.27, F2 = 4.18.
